# Tetra­aqua­bis(5-hydroxy­nicotinato-κ*N*)cadmium(II)

**DOI:** 10.1107/S1600536808035903

**Published:** 2008-11-08

**Authors:** Mei-Xiang Jiang, Yun-Long Feng

**Affiliations:** a Zhejiang Key Laboratory for Reactive Chemistry on Solid Surfaces, Institute of Physical Chemistry, Zhejiang Normal University, Jinhua, Zhejiang 321004, People’s Republic of China

## Abstract

The title compound, [Cd(C_6_H_4_NO_3_)_2_(H_2_O)_4_], was obtained by the reaction of cadmium chloride with 5-hydroxy­nicotinic acid. The Cd^II^ atom is located on an inversion centre and is coordinated by two N atoms from two 5-hydroxy­nicotinic acid ligands and four water mol­ecules in a distorted octa­hedral geometry. The structure is stabilized by inter­molecular O—H⋯O hydrogen bonds, forming a three-dimensional network.

## Related literature

For cadmium componds and their photoluminescent properties, see: He *et al.* (2008 ▶); Kang *et al.* (2007 ▶); Zhang *et al.* (2006 ▶); Zora *et al.* (2006 ▶).
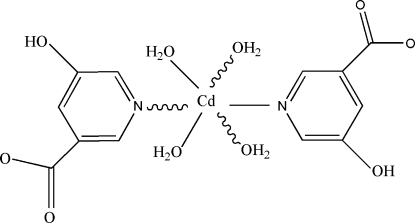


## Experimental

### 

#### Crystal data

[Cd(C_6_H_4_NO_3_)_2_(H_2_O)_4_]
*M* *_r_* = 460.68
Triclinic, 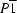

*a* = 7.2190 (1) Å
*b* = 7.2510 (1) Å
*c* = 8.9260 (1) Å
α = 70.377 (1)°
β = 68.154 (1)°
γ = 65.7170 (10)°
*V* = 385.97 (1) Å^3^
*Z* = 1
Mo *K*α radiation
μ = 1.48 mm^−1^
*T* = 296 (2) K
0.27 × 0.17 × 0.07 mm

#### Data collection

Bruker APEXII diffractometer
Absorption correction: multi-scan (*SADABS*; Sheldrick, 1996 ▶) *T*_min_ = 0.667, *T*_max_ = 0.903
6067 measured reflections
1759 independent reflections
1754 reflections with *I* > 2σ(*I*)
*R*_int_ = 0.017

#### Refinement

*R*[*F* ^2^ > 2σ(*F* ^2^)] = 0.016
*wR*(*F* ^2^) = 0.042
*S* = 1.09
1759 reflections
131 parameters
7 restraints
H atoms treated by a mixture of independent and constrained refinement
Δρ_max_ = 0.36 e Å^−3^
Δρ_min_ = −0.34 e Å^−3^

### 

Data collection: *SMART* (Bruker, 2004 ▶); cell refinement: *SAINT* (Bruker, 2004 ▶); data reduction: *XPREP* (Bruker, 2004 ▶); program(s) used to solve structure: *SHELXTL* (Sheldrick, 2008 ▶); program(s) used to refine structure: *SHELXTL*; molecular graphics: *SHELXTL*; software used to prepare material for publication: *SHELXTL*.

## Supplementary Material

Crystal structure: contains datablocks I, global. DOI: 10.1107/S1600536808035903/at2660sup1.cif

Structure factors: contains datablocks I. DOI: 10.1107/S1600536808035903/at2660Isup2.hkl

Additional supplementary materials:  crystallographic information; 3D view; checkCIF report

## Figures and Tables

**Table 1:** Hydrogen-bond geometry (Å, °)

*D*—H⋯*A*	*D*—H	H⋯*A*	*D*⋯*A*	*D*—H⋯*A*
O1*W*—H1*WA*⋯O2^i^	0.81 (2)	1.94 (2)	2.742 (2)	171 (3)
O1*W*—H1*WB*⋯O3^ii^	0.79 (2)	2.20 (2)	2.973 (2)	164 (3)
O2*W*—H2*WA*⋯O1^iii^	0.82 (2)	1.87 (2)	2.656 (2)	160 (3)
O2*W*—H2*WB*⋯O2^iv^	0.82 (2)	1.93 (2)	2.735 (2)	165 (3)
O3—H3⋯O1^v^	0.83 (2)	1.88 (2)	2.664 (2)	157 (3)

Symmetry codes: (i) 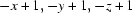; (ii) 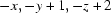; (iii) 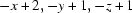; (iv) 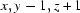; (v) 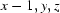.

**Fig. 1.**
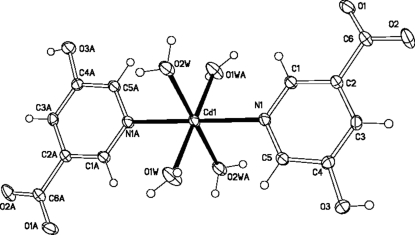
A view of the molecule of (I), showing the atom-labelling scheme, displacement ellipsoids are shown at the 30% probability level. [Symmetry code: (A) -x + 1, -y + 1, -z + 2].

**Fig. 2.**
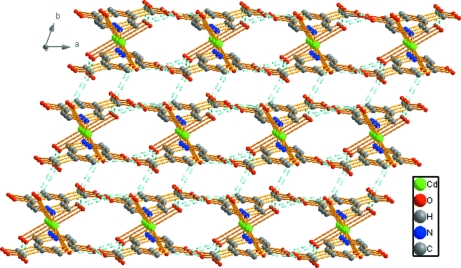
A view of the three dimensional framework of the title compound. The O—H···O interactions are depicted by dashed lines.

## supplementary crystallographic information

### Comment

There is intense research on the synthesis of the cadmium metal compounds for their interesting photoluminescent properties. A large number of these compounds have been synthesized (He *et al.*, 2008; Zora *et al.*, 2006; Kang *et al.*,2007; Zhang *et al.*, 2006).

As illustrated in Fig. 1, the Cd(II) atom is coordinated by two nitrogen atoms from two 5-hydroxynicotinic acid ligands and four water molecules. Four coordinated atoms of O1W, O2W, O1WA and O2WA constitute the base of the octahedral, whereas N1 and N1A atoms occupy the apical position. The intermolecular hydrogen bonds play an important role in the formation of the three-dimensional network. As shown in Fig. 2, the intermolecular O—H···O hydrogen bonds link the neighboring molecules to a three-dimensional network.

### Experimental

A mixture of 0.5 mmol 5-hydroxynicotinic acid and 0.5 mmol of cadmium chloride in 10 ml distilled water was stirred for 30 min at 323 K, then the reaction mixture was filtered and well shaped colourless crystals of the title compound was obtained from the mother liquor by slow evaporation at room temperature for several days.

### Refinement

The H atoms bonded to C atoms were positioned geometrically [aromatic C—H = 0.93 Å and aliphatic C—H = 0.97 Å, *U*_iso_(H) = 1.2*U*_eq_(C)]. The H atoms bonded to O atoms were located in a difference Fourier maps and refined with O—H distance restraints of 0.85 and *U*_iso_(H) = 1.5*U*_eq_(O).

### Crystal data

**Table:**

[Cd(C_6_H_4_NO_3_)_2_(H_2_O)_4_]	*Z* = 1
*M**_r_* = 460.68	*F*_000_ = 230
Triclinic, *P*1	*D*_x_ = 1.982 Mg m^−^^3^
Hall symbol: -P 1	Mo *K*α radiation λ = 0.71073 Å
*a* = 7.21900 (10) Å	Cell parameters from 5615 reflections
*b* = 7.25100 (10) Å	θ = 2.5–27.5º
*c* = 8.92600 (10) Å	µ = 1.48 mm^−^^1^
α = 70.3770 (10)º	*T* = 296 (2) K
β = 68.1540 (10)º	Sheet, colourless
γ = 65.7170 (10)º	0.27 × 0.17 × 0.07 mm
*V* = 385.972 (9) Å^3^	

### Data collection

**Table:**

Bruker APEXII diffractometer	1759 independent reflections
Radiation source: fine-focus sealed tube	1754 reflections with *I* > 2σ(*I*)
Monochromator: graphite	*R*_int_ = 0.017
*T* = 296(2) K	θ_max_ = 27.5º
ω scans	θ_min_ = 2.5º
Absorption correction: multi-scan(SADABS; Sheldrick, 1996)	*h* = −9→9
*T*_min_ = 0.667, *T*_max_ = 0.903	*k* = −9→9
6067 measured reflections	*l* = −11→11

### Refinement

**Table:**

Refinement on *F*^2^	Secondary atom site location: difference Fourier map
Least-squares matrix: full	Hydrogen site location: inferred from neighbouring sites
*R*[*F*^2^ > 2σ(*F*^2^)] = 0.016	H atoms treated by a mixture of independent and constrained refinement
*wR*(*F*^2^) = 0.042	*w* = 1/[σ^2^(*F*_o_^2^) + (0.0249*P*)^2^ + 0.1253*P*] where *P* = (*F*_o_^2^ + 2*F*_c_^2^)/3
*S* = 1.09	(Δ/σ)_max_ < 0.001
1759 reflections	Δρ_max_ = 0.36 e Å^−^^3^
131 parameters	Δρ_min_ = −0.33 e Å^−^^3^
7 restraints	Extinction correction: none
Primary atom site location: structure-invariant direct methods	

### Special details

**Table:**

Geometry. All e.s.d.'s (except the e.s.d. in the dihedral angle between two l.s. planes) are estimated using the full covariance matrix. The cell e.s.d.'s are taken into account individually in the estimation of e.s.d.'s in distances, angles and torsion angles; correlations between e.s.d.'s in cell parameters are only used when they are defined by crystal symmetry. An approximate (isotropic) treatment of cell e.s.d.'s is used for estimating e.s.d.'s involving l.s. planes.
Refinement. Refinement of *F*^2^ against ALL reflections. The weighted *R*-factor *wR* and goodness of fit *S* are based on *F*^2^, conventional *R*-factors *R* are based on *F*, with *F* set to zero for negative *F*^2^. The threshold expression of *F*^2^ > σ(*F*^2^) is used only for calculating *R*-factors(gt) *etc*. and is not relevant to the choice of reflections for refinement. *R*-factors based on *F*^2^ are statistically about twice as large as those based on *F*, and *R*- factors based on ALL data will be even larger.

### Fractional atomic coordinates and isotropic or equivalent isotropic displacement parameters (Å^2^)

**Table:**

	*x*	*y*	*z*	*U*_iso_*/*U*_eq_	
Cd1	0.5000	0.5000	1.0000	0.02626 (6)	
O1	0.8938 (2)	0.7990 (2)	0.32463 (16)	0.0427 (3)	
O1W	0.2684 (3)	0.3168 (3)	1.07212 (17)	0.0482 (4)	
H1WA	0.287 (5)	0.249 (4)	1.009 (3)	0.072*	
H1WB	0.209 (5)	0.270 (4)	1.163 (2)	0.072*	
O2	0.6901 (2)	0.8709 (2)	0.16430 (15)	0.0429 (3)	
O2W	0.7595 (2)	0.2074 (2)	0.92481 (18)	0.0478 (4)	
H2WA	0.881 (3)	0.189 (4)	0.865 (3)	0.072*	
H2WB	0.762 (5)	0.098 (3)	0.994 (3)	0.072*	
O3	0.0066 (2)	0.7675 (2)	0.58671 (17)	0.0400 (3)	
H3	0.002 (4)	0.785 (4)	0.491 (2)	0.053 (7)*	
N1	0.4459 (2)	0.6297 (2)	0.74305 (16)	0.0264 (3)	
C1	0.5945 (2)	0.6740 (2)	0.60554 (19)	0.0267 (3)	
H1A	0.7280	0.6483	0.6138	0.032*	
C2	0.5560 (2)	0.7568 (2)	0.45137 (18)	0.0245 (3)	
C3	0.3595 (3)	0.7885 (2)	0.43778 (19)	0.0263 (3)	
H3A	0.3310	0.8402	0.3354	0.032*	
C4	0.2061 (2)	0.7415 (2)	0.5802 (2)	0.0270 (3)	
C5	0.2549 (2)	0.6655 (3)	0.73035 (19)	0.0276 (3)	
H5A	0.1507	0.6383	0.8260	0.033*	
C6	0.7273 (3)	0.8123 (2)	0.30121 (19)	0.0288 (3)	

### Atomic displacement parameters (Å^2^)

**Table:**

	*U*^11^	*U*^22^	*U*^33^	*U*^12^	*U*^13^	*U*^23^
Cd1	0.02499 (9)	0.03841 (10)	0.01604 (8)	−0.01493 (7)	−0.00552 (6)	−0.00099 (6)
O1	0.0313 (6)	0.0688 (9)	0.0266 (6)	−0.0259 (6)	−0.0010 (5)	−0.0044 (6)
O1W	0.0589 (9)	0.0749 (10)	0.0276 (6)	−0.0484 (8)	−0.0017 (6)	−0.0091 (7)
O2	0.0587 (8)	0.0592 (8)	0.0199 (6)	−0.0385 (7)	−0.0088 (5)	0.0028 (5)
O2W	0.0368 (7)	0.0424 (7)	0.0347 (7)	−0.0075 (6)	0.0072 (6)	0.0013 (6)
O3	0.0285 (6)	0.0620 (8)	0.0326 (7)	−0.0220 (6)	−0.0135 (5)	0.0007 (6)
N1	0.0258 (6)	0.0362 (7)	0.0187 (6)	−0.0141 (5)	−0.0068 (5)	−0.0018 (5)
C1	0.0252 (7)	0.0376 (8)	0.0208 (7)	−0.0155 (6)	−0.0064 (6)	−0.0037 (6)
C2	0.0278 (7)	0.0271 (7)	0.0195 (7)	−0.0128 (6)	−0.0046 (6)	−0.0033 (5)
C3	0.0312 (7)	0.0288 (7)	0.0207 (7)	−0.0120 (6)	−0.0109 (6)	−0.0005 (5)
C4	0.0249 (7)	0.0305 (7)	0.0281 (7)	−0.0112 (6)	−0.0107 (6)	−0.0026 (6)
C5	0.0254 (7)	0.0358 (8)	0.0219 (7)	−0.0143 (6)	−0.0053 (6)	−0.0023 (6)
C6	0.0340 (8)	0.0319 (8)	0.0206 (7)	−0.0166 (6)	−0.0025 (6)	−0.0038 (6)

### Geometric parameters (Å, °)

**Table:**

Cd1—O2W	2.2830 (14)	O3—C4	1.3543 (19)
Cd1—O2W^i^	2.2830 (14)	O3—H3	0.830 (17)
Cd1—N1^i^	2.2831 (13)	N1—C5	1.335 (2)
Cd1—N1	2.2831 (13)	N1—C1	1.3411 (19)
Cd1—O1W	2.3291 (13)	C1—C2	1.387 (2)
Cd1—O1W^i^	2.3291 (13)	C1—H1A	0.9300
O1—C6	1.255 (2)	C2—C3	1.385 (2)
O1W—H1WA	0.809 (17)	C2—C6	1.517 (2)
O1W—H1WB	0.794 (17)	C3—C4	1.389 (2)
O2—C6	1.244 (2)	C3—H3A	0.9300
O2W—H2WA	0.823 (17)	C4—C5	1.386 (2)
O2W—H2WB	0.822 (17)	C5—H5A	0.9300
			
O2W—Cd1—O2W^i^	180.0	C5—N1—C1	118.64 (13)
O2W—Cd1—N1^i^	87.79 (5)	C5—N1—Cd1	117.86 (10)
O2W^i^—Cd1—N1^i^	92.21 (5)	C1—N1—Cd1	123.49 (10)
O2W—Cd1—N1	92.21 (5)	N1—C1—C2	122.29 (14)
O2W^i^—Cd1—N1	87.79 (5)	N1—C1—H1A	118.9
N1^i^—Cd1—N1	180.000 (1)	C2—C1—H1A	118.9
O2W—Cd1—O1W	85.72 (6)	C3—C2—C1	118.97 (14)
O2W^i^—Cd1—O1W	94.28 (6)	C3—C2—C6	121.06 (14)
N1^i^—Cd1—O1W	90.57 (5)	C1—C2—C6	119.96 (14)
N1—Cd1—O1W	89.43 (5)	C2—C3—C4	118.68 (14)
O2W—Cd1—O1W^i^	94.28 (6)	C2—C3—H3A	120.7
O2W^i^—Cd1—O1W^i^	85.72 (6)	C4—C3—H3A	120.7
N1^i^—Cd1—O1W^i^	89.43 (5)	O3—C4—C5	115.78 (14)
N1—Cd1—O1W^i^	90.57 (5)	O3—C4—C3	125.40 (14)
O1W—Cd1—O1W^i^	180.0	C5—C4—C3	118.81 (14)
Cd1—O1W—H1WA	117 (2)	N1—C5—C4	122.55 (14)
Cd1—O1W—H1WB	127 (2)	N1—C5—H5A	118.7
H1WA—O1W—H1WB	110 (2)	C4—C5—H5A	118.7
Cd1—O2W—H2WA	131 (2)	O2—C6—O1	125.02 (15)
Cd1—O2W—H2WB	117 (2)	O2—C6—C2	117.26 (15)
H2WA—O2W—H2WB	105 (2)	O1—C6—C2	117.71 (14)
C4—O3—H3	108.2 (19)		
			
O2W—Cd1—N1—C5	119.15 (12)	C1—C2—C3—C4	1.8 (2)
O2W^i^—Cd1—N1—C5	−60.85 (12)	C6—C2—C3—C4	−177.83 (14)
O1W—Cd1—N1—C5	33.45 (13)	C2—C3—C4—O3	178.81 (15)
O1W^i^—Cd1—N1—C5	−146.55 (13)	C2—C3—C4—C5	0.2 (2)
O2W—Cd1—N1—C1	−61.78 (13)	C1—N1—C5—C4	1.7 (2)
O2W^i^—Cd1—N1—C1	118.22 (13)	Cd1—N1—C5—C4	−179.16 (12)
O1W—Cd1—N1—C1	−147.47 (13)	O3—C4—C5—N1	179.22 (15)
O1W^i^—Cd1—N1—C1	32.53 (13)	C3—C4—C5—N1	−2.1 (2)
C5—N1—C1—C2	0.5 (2)	C3—C2—C6—O2	−5.8 (2)
Cd1—N1—C1—C2	−178.62 (11)	C1—C2—C6—O2	174.61 (15)
N1—C1—C2—C3	−2.2 (2)	C3—C2—C6—O1	173.06 (16)
N1—C1—C2—C6	177.42 (14)	C1—C2—C6—O1	−6.6 (2)

Symmetry codes: (i) −*x*+1, −*y*+1, −*z*+2.

### Hydrogen-bond geometry (Å, °)

**Table:**

*D*—H···*A*	*D*—H	H···*A*	*D*···*A*	*D*—H···*A*
O1W—H1WA···O2^ii^	0.809 (17)	1.941 (17)	2.742 (2)	171 (3)
O1W—H1WB···O3^iii^	0.794 (17)	2.201 (18)	2.9728 (19)	164 (3)
O2W—H2WA···O1^iv^	0.823 (17)	1.867 (18)	2.6556 (18)	160 (3)
O2W—H2WB···O2^v^	0.822 (17)	1.933 (17)	2.7349 (19)	165 (3)
O3—H3···O1^vi^	0.830 (17)	1.878 (19)	2.6637 (19)	157 (3)

Symmetry codes: (ii) −*x*+1, −*y*+1, −*z*+1; (iii) −*x*, −*y*+1, −*z*+2; (iv) −*x*+2, −*y*+1, −*z*+1; (v) *x*, *y*−1, *z*+1; (vi) *x*−1, *y*, *z*.
